# A non-laboratory-based risk score for predicting diabetic kidney disease in Chinese patients with type 2 diabetes

**DOI:** 10.18632/oncotarget.21684

**Published:** 2017-10-09

**Authors:** Mian Wu, Junxi Lu, Lei Zhang, Fengjing Liu, Si Chen, Ying Han, Fangya Zhao, Kaifeng Guo, Yuqian Bao, Haibing Chen, Weiping Jia

**Affiliations:** ^1^ Shanghai Diabetes Institute, Shanghai Key Laboratory of Diabetes Mellitus, Shanghai Clinical Center for Diabetes, Department of Endocrinology and Metabolism, Shanghai Jiaotong University Affiliated Sixth People's Hospital, Shanghai, China; ^2^ Department of Endocrinology and Metabolism, Shanghai Eighth People's Hospital, Shanghai, China

**Keywords:** chinese, diabetic kidney disease, prognosis, risk factors, type 2 diabetes

## Abstract

**Aim:**

To construct a simple screening tool for predicting diabetic kidney disease in Chinese patients with type 2 diabetes.

**Materials and Methods:**

In the development cohort, the clinical and procedural characteristics of the 4,795 patients were considered as candidate univariate predictors of diabetic kidney disease. The β-coefficients derived from a multiple logistic regression model predicting the presence of DKD were used to calculate the risk score. The performance of the risk score was validated in a cross-sectional and a prospective cohort population.

**Results:**

The risk score included sex, body mass index, systolic blood pressure, and duration of diabetes. The total point ranged from 0 to 39. In the development cohort, compared with participants with risk score < 10, those with risk score between 10 to 20, 21 to 30, and > 30 had ORs of 3.21, 7.92 and 17.55 for developing diabetic kidney disease, respectively. In the prospective cohort, 60.9% patients with risk score over 30 were expected to develop DKD at 72 months of follow-up.

**Conclusions:**

Sex, body mass index, systolic blood pressure, and duration of diabetes were independent predictors of diabetic kidney disease, and the derived risk equation was a simple screening tool for screening diabetic kidney disease in Chinese patients with type 2 diabetes.

## INTRODUCTION

Diabetic kidney disease (DKD) is a major complication of diabetes and is the leading cause of end-stage renal failure (ESRD) worldwide [1]. One study projected that 113.9 million persons in China are affected by diabetes [2]. Extrapolations from cross-sectional studies have found that micro- or macroalbuminuria affects up to 60% of Asian patients, and it is possible that some 68 million persons with diabetes in China have DKD [3], which places a tremendous burden on the healthcare system. Early detection of and intervention in DKD may reduce exposure to long-term kidney dysfunction and prevent or delay ESRD.

China, a developing country, has lots of remote areas which economic development is less developed and lacks of medical care. As a result of the limitation of economic and medical conditions in remote areas, it is impossible for every diabetic patient to screen for DKD, which needs to check fundus and microalbuminuria. Screening DKD on those patients with high-risk of DKD can reduce cost, improve screening efficiency, and improve the efficiency of the health economics. However, practical, readily applicable methods to assess the DKD risk in patients with type 2 diabetes have not been specifically developed.

Risk score developed based on demographic, anthropometric, and clinical information without a laboratory test has proven useful and inexpensive as a stepwise screening strategy for undiagnosed type 2 diabetes [4–7]. This approach is particularly useful in China, considering the large population and already high and still increasing prevalence of undiagnosed DKD. Thus, a simple risk score for DKD screening in Chinese patients with type 2 diabetes is urgently needed.

This study developed a non-laboratory based risk score according to the definition of DKD which released by the National Kidney Foundation and Kidney disease Outcomes Quality Initiative (NKF-KDOQI) [8], in a cross-sectional cohort and further validated in a cross-sectional and a prospective cohort.

## MATERIALS AND METHODS

### Study design and population

**Figure 1:**
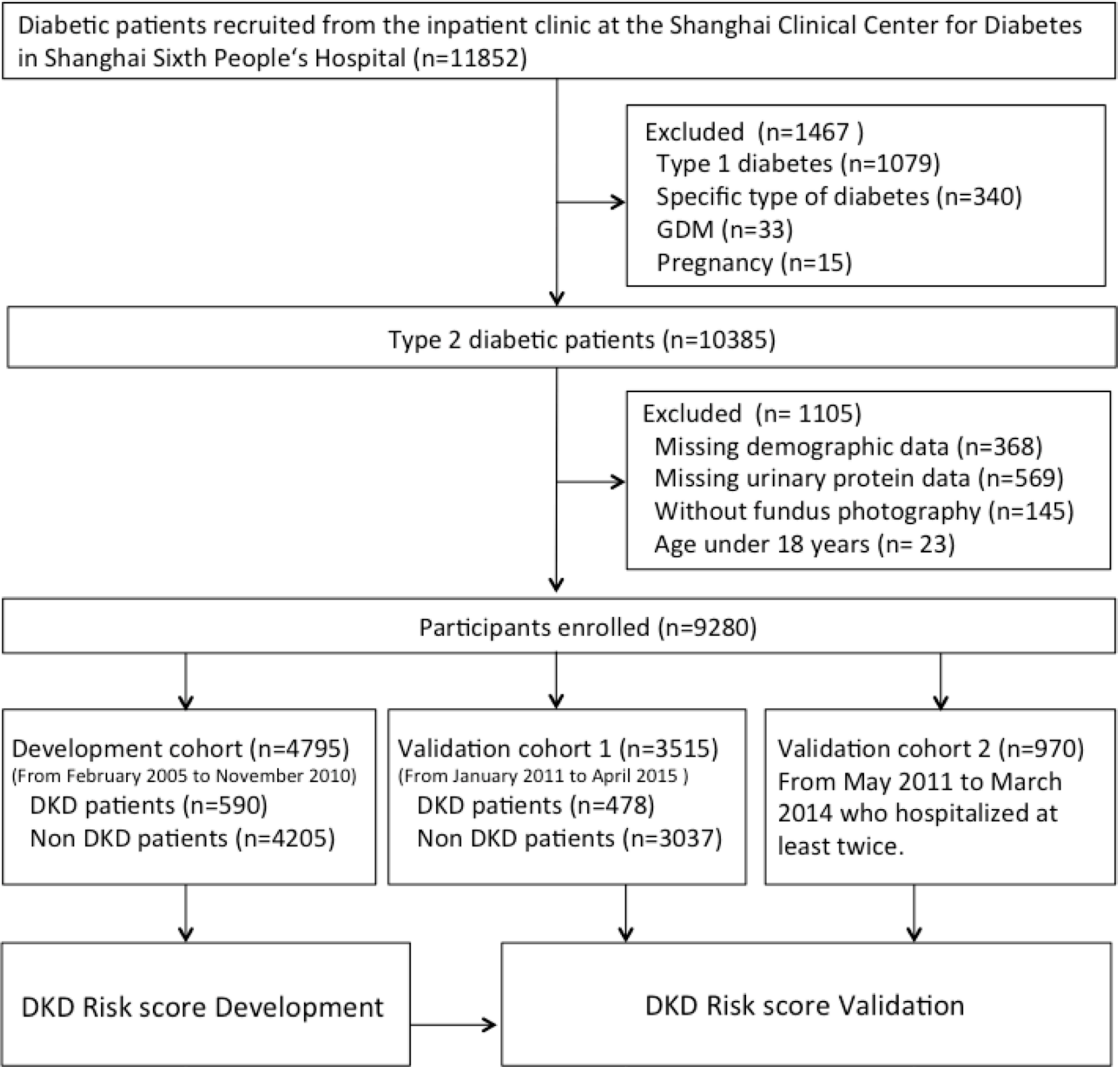
Flow diagram of recruitment of participants GDM = gestational diabetes mellitus, DKD = diabetic kidney disease.

All patients were recruited from the inpatient clinic of the Shanghai Clinical Center for Diabetes in Shanghai Sixth People's Hospital (Shanghai, China). They completed a uniform questionnaire containing of questions about their histories of current and previous illnesses and medical treatment. Exclusion criteria were cancer, severe psychiatric disturbance, chronic kidney disease, pregnancy, and glucocorticoid treatment. Type 2 diabetes was diagnosed based on the American Diabetes Association guidelines (2006). Diabetic kidney disease was diagnosed according to the criteria of the NKF-KDOQI (2007) [4]. After excluding 368 individuals with missing demographic data, 569 with missing urinary protein data, 145 without fundus photography, and 23 aged < 18 years, 9,280 patients were enrolled in the study. A total of 4,795 participants recruited from February 2005 to November 2010 were included in the development cohort, 3,515 patients enrolled from January 2011 to April 2015 were included in validation 1, and 970 patients with type 2 diabetes mellitus without DKD who were hospitalized at least twice were recruited for validation 2 (Figure 1). In validation 2 population, the first and last hospitalization information was used for analysis (median observation period 2.7 years, interquartile range 1.6–4.2 years). All studies were approved by the Ethics Committee of Shanghai Jiao Tong University Affiliated Sixth People's Hospital, and informed consent was obtained from all participants.

The demographic and clinical data of the subjects were recorded, including age, sex, diabetes duration, height, weight, waist circumference, waist circumference, systolic blood pressure (SBP), diastolic blood pressure (DBP). Weight was measured in kilograms and height in meters. The body mass index (BMI) was calculated as weight in kilograms divided by height in meters squared. Waist and hip circumference was measured in centimeter. The waist and hip ratio (WHR) was calculated as waist circumference divided by hip circumference. SBP and DBP were recorded at 1-minute intervals after a minimum of 5 minutes rest and calculated as the mean of the last two of three measurements. Venous blood samples were collected in the morning after an overnight fast to measure blood glucose, HbA1c, insulin, C-peptide, electrolytes, and lipid profiles. Participants were asked to provide 24-hour urine samples. A maximum of three 24-hour urine samples were collected from each patient to detect urinary protein. Glomerular filtration rate (GFR) was calculated using the Modification of Diet in Renal Disease equation. Fundus photography was performed by experienced physician. All subjects underwent standard clinical and laboratory evaluations.

### Risk score development

The risk score development dataset was initially used to identify univariate associations between the baseline clinical and key procedural characteristics and DKD. Multivariate logistic regression analysis was then performed to identify independent predictors of DKD and to estimate odds ratios (ORs). Risk factors that were significant in the univariate analyses were available for selection in the final model; a bootstrap method was used to select the best subset of risk factors to avoid over-fitting the data. From the development dataset, 1000 bootstrap samples were selected. For each sample, a stepwise selection procedure was used to choose independent predictors of DKD. Variables that were selected in at least 90% of the bootstrap models were included in the final multivariate models. All candidate risk factors were categorized. The DKD Risk Score was derived by multiplying the β-coefficients of the significant variables by 10 and rounding to the nearest integer. The final risk score represented the sum of the integer coefficients. According to the final risk score quartile, patients were categorized into relatively low, moderate, high, and very high groups. The Hosmer–Lemeshow test was used to investigate how close the prevalence predicted by the multivariate model was to the observed prevalence. The difference was considered non-significant at *P* > 0.05. The optimal cutoff point was identified using the Youden index, which was at the maximum sum of the sensitivity and specificity –1.

### Risk score validation

The DKD Risk Score developed from the exploratory population was externally validated in two cohorts: a cross-sectional cohort (validation 1) and a prospective cohort (validation 2). In validation 1, we used the developed risk score to calculate the total score of each subject, and then predicted the probability of incidence of DKD by regression analysis. The predicted incidence of DKD and the actual incidence of DKD were used to construct the receiver operating characteristic (ROC) curve. The ROC curve was obtained by plotting sensitivity against (1 – specificity) at each cutoff value. Diagnostic accuracy was assessed by the area under the curve (AUC). In validation 2, type 2 diabetic patients without DKD were recruited. Baseline variables were used to calculate the total score according to the developed risk score; however, follow-up DKD incidence instead of baseline data was used to calculate the area under ROC curve. C statistics were used to compare the AUCs. The difference between AUCs was examined using the Mann–Whitney *U*-test.

To validate the predictive power of the risk score, patients from validation 2 were classified by risk stratification, and Kaplan–Meier curves were generated. Differences between two groups were assessed using the log-rank test. The statistical analysis was performed using SPSS 21.0 (SPSS, Chicago, IL).

## RESULTS

### Characteristics of the study population

The development population had better glycemic control and a longer duration of diabetes compared with the validation 1 and validation 2 samples. The development and validation 2 populations had a lower blood lipid level, higher GFR, and lower proportion of men compared with the validation 1 population (Table 1). In the development dataset, the DKD group was more obese and had a higher blood glucose level, more severe hypertension, and a higher proportion of males than the non-DKD group (Table 2).

**Table 1:** Baseline characteristics of participants in the development population, validation 1 and validation 2

	Development population	Validation 1	Validation 2^a^
N (male%)	4795 (56.2)	3515 (51.0)	970 (55.4)
Age (years)	59.4 ± 12.3	59.5 ± 12.4	58.2 ± 12.3
BMI (kg/m^2^)	25.1 ± 3.6	25.0 ± 3.5	25.2 ± 3.5
WHR	0.93 ± 0.07	0.92 ± 0.06	0.92 ± 0.07
Systolic blood pressure (mmHg)	132.5 ± 16.8	132.2 ± 17.6	130.3 ± 16.0
Diastolic blood pressure (mmHg)	79.8 ± 9.4	80.0 ± 9.6	79.7 ± 9.4
FPG (mmol/L)	7.9 ± 2.8	8.4 ± 2.9	8.4 ± 2.9
2-h PG (mmol/L)	12.8 ± 4.4	14.0 ± 5.1	13.8 ± 4.6
HbA_1c_ (%) (mmol/mol)	8.6 ± 2.0 (70.5 ± 21.9 )	9.1 ± 2.3 (80.0 ± 25.1)	8.9 ± 2.5 (73.8 ± 27.3)
TG (mmol/L)	1.74 ± 1.60	1.92 ± 1.87	1.91 ± 1.79
TC (mmol/L)	4.71 ± 1.18	4.77 ± 1.14	4.71 ± 1.06
Family history of diabetes (%)	54.1	/	54.8
Duration of diabetes (years)	10.2 ± 7.2	7.9 ± 6.8	8.2 ± 6.6
ACR (mg/mmol)	264 ± 822	127 ± 540	98 ± 213
GFR (ml/min)	94.8 ± 24.9	89.1 ± 32.1	97.1 ± 24.4
DKD (%)	12.3	13.6	10.1^b^

Data are mean ± SD or n (%), unless otherwise indicated. ^a^ Baseline characteristics unless otherwise indicated. ^b^ Follow-up data.

**Table 2:** Comprasion of non-DKD group and DKD group in development population

	Non-DKD	DKD	*P*
N (male%)	4416 (55.7%)	643 (60.3%)	< 0.001
Age (years)	59.3 ± 12.5	60.0 ± 11.3	0.384
BMI (kg/m^2^)	25.0 ± 3.6	26.1 ± 3.7	< 0.001
WHR	0.93 ± 0.07	0.95 ± 0.06	< 0.001
Systolic blood pressure (mmHg)	131.0 ± 15.8	142.6 ± 19.5	< 0.001
Diastolic blood pressure (mmHg)	79.3 ± 9.0	83.4 ± 11.2	< 0.001
FPG (mmol/L)	7.9 ± 2.8	8.3 ± 3.2	0.012
2-h PG (mmol/L)	12.7 ± 4.3	12.8 ± 4.8	0.619
HbA_1c_ (%) (mmol/mol)	8.5 ± 2.1 (69.4 ± 23.0)	8.9 ± 2.1(73.8 ± 23.0)	< 0.001
TG (mmol/L)	1.67 ± 1.47	2.25 ± 2.20	< 0.001
TC (mmol/L)	4.66 ± 1.13	5.09 ± 1.42	< 0.001
Family history of diabetes (%)	53.8	56.5	0.086
Duration of diabetes (years)	9.6 ± 7.0	12.4 ± 7.1	< 0.001
ACR (mg/mmol)	38.5 ± 85.2	1280 ± 1492.2	< 0.001
GFR (ml/min)	96.6 ± 24.1	82.7 ± 27.1	< 0.001

### Generation of DKD risk score

The purpose of this study was to explore a risk score model that predicts DKD in Chinese type 2 diabetic patients as simply as possible. Although univariate analyses showed that many variables were significantly associated with DKD, including demographic characteristics (male gender), obesity-related indicators (BMI, WHR, and hyperlipidemia), glycemia control (fasting plasma glucose, fasting C peptide, and HbA_1c_), hypertension, and duration of diabetes, we used only variables that can could be measured or obtained easily for further analysis.

**Figure 2:**
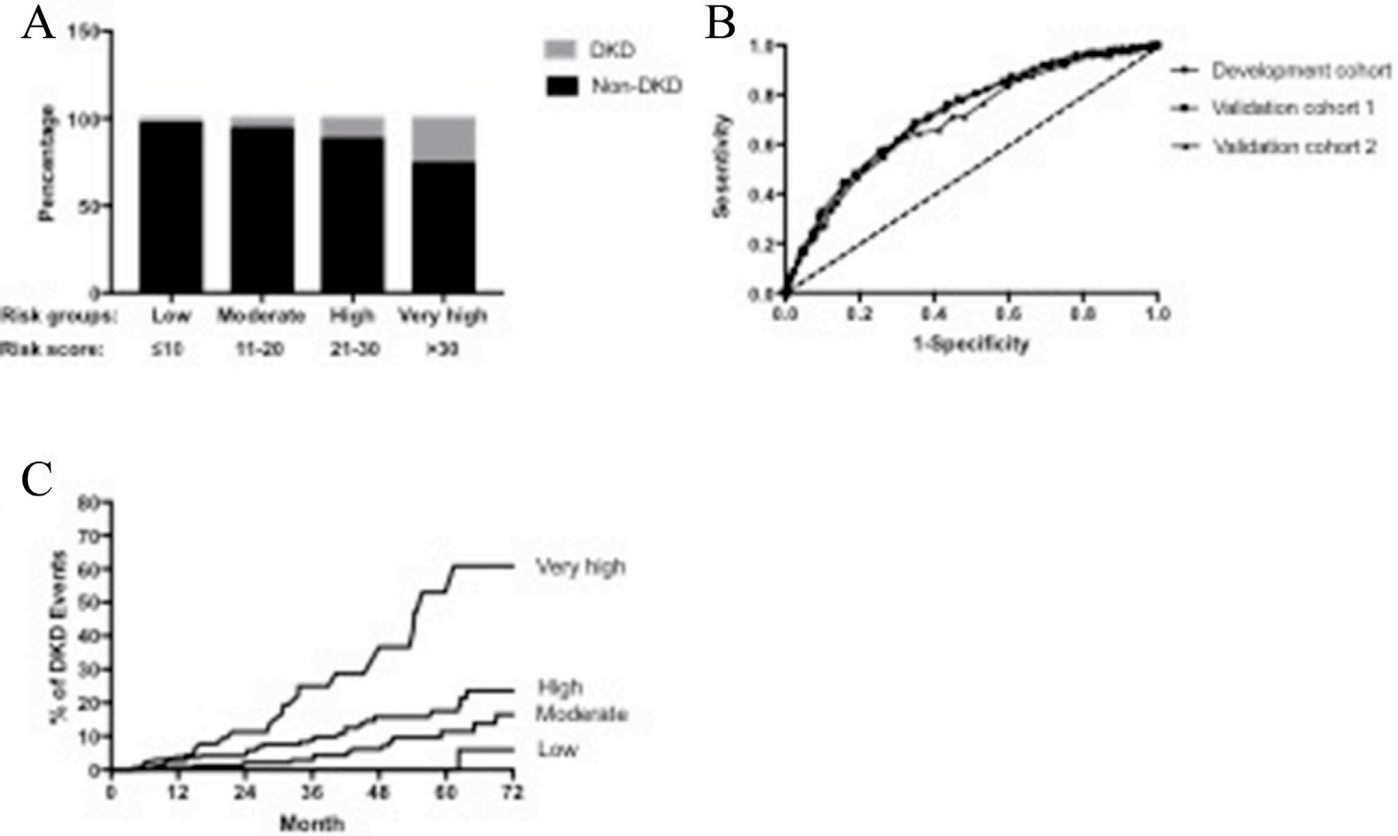
(**A**) Prevalence of DKD in four risk groups stratified by risk score in the development population. (**B**) Receiver operating curve (ROC) for the development, validation 1 and validation 2 cohorts. The AUC of development, validation1 and validation 2 were 0.713(0.692–0.734), 0.720 (0.696–0.744) and 0.696(0.632–0.760), respectively. Difference between the curves for DKD was not significant (U = 0.43, *P* = 0.667). (**C**) Kaplan-Meier curve of DKD end point for each risk group. Moderate group: HR 2.04 (95% confidence interval [CI] 0.76 to 5.46), *P* = 0.23. High group: HR 3.97 (95% CI 1.90 to 8.33), *P* = 0.01. Very high group: Hazard ratio (HR) 11.37 (95% CI 4.76 to 27.19), *P* < 0.0001. Low risk group: Reference.

In the multivariate logistic regression analysis, male sex, BMI, systolic blood pressure, and duration of diabetes were independently significantly associated with the presence of DKD. The Hosmer–Lemeshow test showed that the predicted prevalence of DKD in type 2 diabetes patients in the multivariable model matched the observed prevalence well (*x*^2^ = 9.379, *P* = 0.310). The risk score was developed based on the multivariate model (Table 3). The point total ranged from 0 to 39. The AUC of the ROC curve was 0.713 (95% CI 0.692–0.734) in the development population. Based on the obtained frequencies of DKD using different risk scores, the 4,795 patients were further categorized into four groups: low (*n* = 484), moderate (*n* = 1,666), high (*n* = 1,888), and very high (*n* = 721) risk groups, corresponding to risk scores of < 10, 10–20, 21–30, and > 30, respectively. The incidences of DKD in those four groups were 2.1, 6.7, 15.2, and 28.4%, respectively (Figure 2A). Compared with participants in low group, those with in moderate, high and very high groups had ORs of 3.21 (95% CI 1.76–5.88), 7.92 (4.41–14.22) and 17.55 (9.68–31.80) for developing DKD, respectively (*P* < 0.001).

**Table 3:** Odds ratio (95% CI) and β-coefficient for prevalence of DKD in the 4,795 participants of the development population, estimated using logistic regression analysis

	β-Coefficient	OR (95% CI)	Score
Sex			
Women			0
Men	0.525	1.690 (1.406–2.032)	5
BMI (kg/m^2^)			
< 25			0
25–27.99	0.322	1.380 (1.124–1.695)	3
≥ 28	0.602	1.825 (1.459–2.283)	6
Systolic blood pressure (mmHg)			
< 120			0
120–129	0.626	1.870 (1.190–2.939)	6
130–139	0.970	2.638 (1.696–4.102)	10
≥ 140	1.732	5.651 (3.705–8.617)	17
Duration of diabetes (years)			
< 5			0
5–9.9	0.322	1.380 (1.014–1.878)	3
10–14.9	0.794	2.211 (1.668–2.932)	8
≥ 15	1.074	2.928 (2.230–3.845)	11

According to the Youden index, the optimal cutoff risk score was 20. At the cutoff point ≥ 20, the sensitivity and specificity of the DKD Risk Score were 80.3% and 49.0%, respectively. The positive and predictive values were 16.1% and 96.5%, respectively.

### Validation of DKD risk score

The AUCs of the Chinese DKD Risk Score for detecting the prevalence of DKD in patients with type 2 diabetes based on validation 1 and validation 2 were 0.720 (95% CI 0.696–0.744) and 0.696 (0.632–0.760), respectively. No significant differences were found between the ROC area of the development and the validation cohorts (U = 0.43, *P* = 0.667) (Figure 2B).

Figure 2C shows the Kaplan–Meier curves for DKD grouped by risk score. Compared with the low-risk group, high and very high groups had significantly higher hazard ratios. Compared with patients with risk score < 20, the risk of developing DKD in patients with risk score ≥ 20 increased 11.4-fold. Time-varying covariate analyses showed that at 36 months, approximately 16.2% of the patients in the very-high group had developed DKD (i.e., for a hypothetical cohort of patients who remained in the very –high group throughout the trial, 16.2% would be expected to develop DKD within 36 months). For patients in low, moderate and high group, 0, 2.1, and 6.9% developed DKD, respectively. At 72 months, 5.9, 16.4, 23.6 and 60.9% were expected to develop DKD in low, moderate, high and very high group, respectively.

In total, 1685 (47.9%) individuals in validation 1 and 462 (47.6%) in validation 2 had a risk score ≥ 20 points. At the cutoff point ≥ 20, the sensitivity and specificity were 76.0 and 56.6% in validation 1 and 71.2 and 55.0% in validation 2, respectively.

## DISCUSSION

This study proposed a DKD Risk Score based on four readily available variables and showed that a higher score confers an exponentially increased DKD risk. The performance of the Chinese DKD Risk Score is adequate for detecting and predicting DKD in Chinese type 2 diabetic patients.

Several risk factors for predicting [9–15] or detecting [16–20] DKD in type 2 diabetes have been identified; most of these were derived from Caucasian populations [9, 14, 20], and only a few have been based on Asian populations [15, 18]. The common independent risk factors are blood pressure and renin-angiotensin-aldosterone system inhibitor use, glycemic control, and anthropometric and laboratory indicators of obesity. In our study, independent risk factors for the development of DKD were male gender, hypertension, obesity, and increased duration of diabetes, demonstrating that these traditional risk factors are important in patients with DKD.

Among the four risk factors, SBP was the most powerful baseline risk factor for detecting of DKD. Hypertensive individuals had a 5.651-fold increase in the risk of developing DKD in this study. Consistent with our finding, the central importance of blood pressure as a risk factor for both albuminuria and renal impairment in type 2 diabetes has been well documented in observational studies [12, 17]. Control of hypertension is of primary importance in patients with diabetic nephropathy. Antihypertensive drugs, such as angiotensin-converting enzyme inhibitors and angiotensin receptor blockers have been documented to delay the progression of DKD by preventing the incidence of albuminuria, reducing the microalbuminuria level, and preserving renal function [21–24].

After blood pressure, the duration of diabetes was the second most powerful risk factor for DKD in type 2 diabetic patients in our study. Compared with participants with a duration of diabetes < 5 years, those with a duration of diabetes ≥ 15 years had an OR of 2.928 for developing DKD. A previous study also reported that the duration of diabetes were risk factors for overt nephropathy and microalbuminuria in urban Asian Indians [19]. In the ADVANCE trial, diabetes duration was associated with the risk of microvascular events (1.28 [1.23–1.33]) in 11,140 patients with type 2 diabetes [25].

Male gender has been associated with the development of nephropathy in diabetes in many studies. In our study, males had a higher frequency in the total sample, but the frequency was the highest in the DKD group, accounting for 63.3% of the development cohort. In a prospective observational study involving 176 patients with type 2 diabetes, Gall *et al*. [11] found that males had a 2.6 times greater risk of developing incipient or overt nephropathy.

The prevalence of obesity has risen to epidemic proportions and continues to be a major health problem worldwide [26]. There are clear associations between BMI and visceral obesity and renal dysfunction. Data from our study demonstrated a 1.825-fold increase in the odds of developing DKD in subjects with a high BMI (≥ 28 kg/m^2^) compared with those with normal weight. Many studies suggest that obesity is a risk factor for ESRD and chronic kidney disease (CKD). In a cohort of over 11,000 apparently healthy men followed for 14 years, a higher baseline BMI was associated with an increased risk of incident CKD. Compared with participants with a BMI < 22.7 kg/m^2^, those with a BMI > 26.6 kg/m^2^ had an OR of 1.45 for developing CKD, and those who experienced a BMI increase of > 10% had an increased risk of CKD (OR = 1.27) [27].

Our findings emphasize the importance of SBP, BMI, and an increased duration of diabetes for predicting the development of DKD in male type 2 diabetic patients. Compared with previous studies, we weighed these risk factors and calculated the cumulative risk due to their combination. The DKD Risk Score had good discriminative power with an AUC of 0.713 in the development population, 0.720 in the validation 1 sample, and 0.696 in the validation 2 sample. No significant differences were found between the ROC area of the development and the validation cohorts, which represented that the risk score was available for not only detecting but also predicting DKD in type 2 diabetic patients. Furthermore, by calculating risk score, it was easy to distinguish high-risk patients from other populations. In the development cohort, compared with participants in low risk group, those in moderate, high and very high groups had 3.21, 7.92 and 17.55-fold increase in the odds of developing DKD, respectively. In the prospective cohort, compared with patients with risk score ≤ 20, the risk of developing DKD in patients with risk score > 20 increased 11.4-fold and 60.9 % patients in very high risk group was expected to develop DKD at 72 months of follow-up.

Importantly, our DKD Risk Score does not include a variable that requires blood sampling and can be used by all healthcare providers, including those in remote areas with scarce medical resources, to increase the ability to screen DKD in type 2 diabetes patients. For people who can measure blood pressure at home, the risk score can also be determined by lay populations. Furthermore, this proposed simple risk score for DKD allows for immediate identification of the variables of interest and the identification of high-risk populations. This is particularly important because DKD is seldom reversible, and appropriate, timely treatment including medication and life-style interventions would delay the progress of the disease and reduce the personal and national financial burden, which are important and meaningful.

This study has several limitations. First, previous studies showed that smoking was a risk factor for DKD [28–29], however, since the data on smoking in this study was incomplete, we wasn't able to analyze the effect of smoking on DKD. Second, the sample size was moderate, and the observation period was not long. Third, patients with type 2 diabetes mellitus were recruited from a single hospital, and the cohort was clinic-based rather than population-based. Therefore, our cohort may not be fully representative, and care should be taken when the risk score is used in other Chinese diabetic populations. Fourth, most existing risk scores performed better in their original population than in other validation populations, implying that a race- or country-specific risk score is needed. In the current study, the Chinese DKD Risk Score was validated in two Chinese populations living in Shanghai; in view of the considerable diversity of the Chinese population, further validation is essential before our results can be put to clinical use. Fifth, the Chinese DKD Risk Score had a low specificity at the optimal cutoff value. However, the sensitivity and specificity trade off with each other. The choice of a cutoff value depends on the purpose of applying the risk score. As an effective, inexpensive health-promotion tool instead of a diagnostic test, the risk score can reach a large lay population quickly via the media, Internet, and primary care clinics. As a consequence of the widespread use of this risk score, public awareness of DKD and the factors that bear on the risk score should be increased significantly. From a public health perspective, a high sensitivity is desired.

In conclusion, despite these limitations, based on data from a cross-sectional cohort of Chinese type 2 diabetic patients with DKD, we developed a simple risk score including gender, SBP, BMI, and duration of diabetes for predicting DKD with high accuracy. Given the rising burdens of type 2 diabetes and DKD, especially among Asian populations, including the Chinese, validation of the equation in other populations could make an important contribution to public health.
